# Bio-efficacy of field aged novel class of long-lasting insecticidal nets, against pyrethroid-resistant malaria vectors in Tanzania: A series of experimental hut trials

**DOI:** 10.1371/journal.pgph.0002586

**Published:** 2024-10-04

**Authors:** Jackline L. Martin, Louisa A. Messenger, Mark Rowland, Franklin W. Mosha, Edmund Bernard, Monica Kisamo, Shaban Limbe, Patric Hape, Charles Thickstun, Crene Steven, Oliva Moshi, Boniface Shirima, Nancy S. Matowo, Jacklin F. Mosha, Dominic P. Dee, Thomas S. Churcher, Manisha A. Kulkarni, Alphaxard Manjurano, Natacha Protopopoff

**Affiliations:** 1 Department of Parasitology, Pan-African Malaria Vector Research Consortium, Kilimanjaro Christian Medical University College, Moshi, United Republic of Tanzania; 2 Department of Parasitology, National Institute for Medical Research, National Institute for Medical Research- Mwanza Center, Mwanza, United Republic of Tanzania; 3 Department of Disease Control, Faculty of Tropical Diseases, London School of Hygiene and Tropical, London, United Kingdom; 4 Department of Environmental and Occupational Health, School of Public Health, University of Nevada, Las Vegas, Nevada, United States of America; 5 School of Epidemiology and Public Health, University of Ottawa, Ottawa, ON, Canada; 6 Imperial College London, London, United Kingdom; Malaria Research and Training Center, University of Science, Techniques and Technology of Bamako, MALI

## Abstract

New classes of long-lasting insecticidal nets (LLINs), have been recommended by the World Health Organization (WHO) to control malaria vectors resistant to pyrethroid insecticides. This study was nested in a large-scale cluster-randomized controlled trial conducted (cRCT) in Tanzania. A series of experimental hut trials (EHTs) aimed to evaluate the bio-efficacy of trial LLINs on mosquito indicators most pertinent to malaria transmission over 3 years of use in the community in order to better understand the outcomes of the cRCT. The following field-collected LLINs were assessed: 1) Olyset Plus (combining piperonyl butoxide synergist and permethrin), 2) Interceptor G2 (chlorfenapyr and alpha-cypermethrin), 3) Royal Guard (pyriproxyfen and alpha-cypermethrin), 4) Interceptor (alpha-cypermethrin only) conducted in parallel with 5) a new Interceptor, and 6) an untreated net. Thirty nets of each type were withdrawn from the community at 12, 24, and 36 months after distribution and used for the EHTs. Pre-specified outcomes were 72-hour mortality for Interceptor G2, 24-hour mortality for Olyset Plus, and fertility based on egg development stage for Royal Guard. Overall, Interceptor G2 LLINs induced higher 72-hour mortality compared to standard LLINs of the same age up to12 months (44% vs 21%, OR: 3.5, 95% CI: 1.9–6.6, p-value < 0.001), and 24-hour mortality was only significantly higher in Olyset Plus when new (OR: 13.6, 95%CI: 4.4–41.3, p-value < 0.001) compared to standard LLINs but not at 12 months (17% vs 13%; OR: 2.1, 95% CI: 1.0–4.3; p-value = 0.112). A small, non-significant effect of pyriproxyfen on *Anopheles* fertility was observed for Royal Guard up to 12 months (75% vs 98%, OR: 1.1, 95% CI: 0.0–24.9, p-value = 0.951). There was no evidence of a difference in the main outcomes for any of the new classes of LLINs at 24 and 36 months compared to standard LLINs. Interceptor G2 LLINs showed superior bio-efficacy compared to standard LLINs only up to 12 months, and the effect of Olyset Plus was observed when new for all species and 12 months for *An*. *gambiae* s.l. only. The pyriproxyfen component of Royal Guard had a short and limited effect on fertility. The decrease in effectiveness of Olyset Plus and Royal Guard LLINs in the EHTs aligns with findings from the cRCT, whereas efficacy of Interceptor G2 lasted for a longer period in the cRCT compared to the EHT. Further investigations are needed to understand the complete scope of chlorfenapyr mode of action. Additional EHT in various contexts will help confirm the residual efficacy of the dual active ingredient LLINs and support the development of longer-lasting nets.

## Introduction

Malaria persists as a significant global challenge, with an estimated 241 million cases and 627,000 deaths in 2020. The African region, contributes to 96% of all malaria cases and associated fatalities [1]. In an effort to alleviate the malaria burden, 2.3 billion insecticide-treated nets, specifically long-lasting insecticidal nets (LLINs), were distributed globally between 2004 and 2022, with a substantial 86% delivered to sub-Saharan Africa [1]. In 2020, malaria endemic countries received an estimated 229 million LLINs, with 19.4 million of them being pyrethroid-piperonyl butoxide (PBO)-treated nets [1]. LLINs and other vector control interventions are predicted to have averted 1.7 billion malaria cases and 10.6 million deaths between 2000 and 2020 [1]. Coincident with the scaling up of LLINs and other insecticidal tools across sub-Saharan Africa, mosquito vector populations have developed resistance to pyrethroids, which until recently were the only insecticide class recommended for use in LLINs. Despite the physical and chemical barrier provided by intact LLINs, offering a high degree of personal protection even against resistant mosquitoes, the development of holes or a decline in insecticide efficacy with net ageing could confer a significant competitive advantage to resistant mosquitoes [2]. Multiple studies have illustrated a diminishing effectiveness of LLINs in eliminating mosquitoes in regions characterized by high pyrethroid resistance [3, 4]. As a results reduction of malaria has come to a halt due to the emergence of both biological and non-biological challenges. This has led to a swift increase in insecticide resistance across all *Anopheles* species [5, 6], and poses a significant threat to the efficacy of vector control interventions. The influence of insecticide resistance on malaria transmission varies across different geographical locations [7, 8].

To tackle the spread of resistance, new malaria vector control tools were needed. The first new class of LLINs developed to control resistant mosquitoes was a combination net containing a pyrethroid insecticide and the synergist PBO [9]. In experimental field trials, these LLINs induced significant mortality in vector species [10]. The study done in Burkina Faso reported Olyset Plus outperformed pyrethroid-only LLIN [11]. This class of LLINs was also evaluated in a cluster-randomized control trial (cRCT) in Tanzania and Uganda. In Tanzania, the study found 44% reduction malaria prevalence in the pyrethroid-PBO LLIN arm (Olyset Plus brand) compared to the standard pyrethroid LLIN arm (Olyset net) after one year and a 33% reduction after two years [12]. In a Ugandan cluster-randomized controlled trial (cRCT) conducted over 18 months, the pyrethroid-PBO LLINs (PermaNet 3.0 and Olyset Plus) demonstrated a 13% and 14% reduction in parasite prevalence after 12 and18 months respectively. These findings prompted the World Health Organization (WHO) to acknowledge the public health significance of pyrethroid-PBO LLINs and issue a conditional recommendation for their use as a novel class of vector control product [13].

Since then, two additional dual- active ingredient (AI) LLINs (Royal Guard and Interceptor G2) have undergone evaluation in WHO Phase I and II trials, showing significant promise compared to standard LLINs in combating pyrethroid-resistant vectors. In Phase I studies, Royal Guard (containing the pyrethroid alpha-cypermethrin, and the juvenile growth hormone inhibitor pyriproxyfen) met the WHO criteria with 95% knockdown and more than 80% mortality for up to 20 washes when susceptible *Anopheles (An*.*) gambiae* s.s. (kisumu strain) were exposed in cone assays [14]. It also met the WHO criteria in tunnel tests, with mortality exceeding 80% after 20 washes using the same strain. In a Phase II experimental hut trial conducted against wild, pyrethroid resistant *An*. *gambiae* s.l., Royal Guard demonstrated an 83% reduction in oviposition and a 95% reduction in offspring/hatching before washing. These values decreased to 25% and 50%, respectively, after 20 washes. Interceptor G2 (containing alpha-cypermethrin and the pyrrole chlorfenapyr) was able to induce 71% mortality against free flying *An*.*gambiae* s.l. in an experimental hut trial compared to an alpha-cypermethrin-only net (20% mortality) [15]. In a large-scale cRCT conducted in Tanzania, Royal Guard, Interceptor G2 and Olyset Plus LLINs were evaluated against standard Interceptor nets in the context of resistant malaria vectors. The results revealed 55% lower odds of malaria infection in children aged 6 to 14 years and 44% reduction in malaria case incidence in children aged 6 to 10 years after two years of net use in the Interceptor G2 arm. Additionally, the entomological inoculation rate (EIR) was also significantly lower (by 85%) in the Interceptor G2 arm compared to the standard pyrethroid-only arm [16]. In the same trial, Olyset Plus showed a shorter protective effect of 12 months compared to a previous cRCT conducted in a different part of Tanzania, where pyrethroid-PBO LLINs remained effective for 24 months [12, 16]. A second cRCT conducted in Benin showed similar results, with Interceptor G2 reducing malaria incidence by 46% to children aged 6 to 10 years compared to pyrethroid-only LLINs, while Royal Guard did not significantly reduce malaria outcomes [17]. Based on evidence from these two trials, Interceptor G2 received a strong recommendation from WHO, while Royal Guard received a conditional recommendation. For Royal Guard, a third trial conducted in Burkina Faso [18] was considered by WHO. It was the only trial showing a small but significant reduction in malaria outcomes in the PPF arm compared to the standard LLIN arm.

New products must also undergo WHO pre-qualification to demonstrate efficacy in phase II controlled semi field evaluation as well as phase III community study to assess efficacy over the intended life span of the product (3 years for LLIN) in normal condition of use.

During the Phase II semi field evaluations of LLINs efficacy, it is anticipated that each net should maintain insecticidal activity through at least 20 standardized washes, considering vector knock-down, mortality, and blood feeding inhibition as per WHO guidance [19]. Standard phase II studies are conducted using unwashed nets and nets being washed 20 times as a proxy for a 3-year net’s use in the community. Olyset Plus, Royal Guard and Interceptor G2 have all completed the standard phase II studies and have received temporary pre-qualification [20].

Alongside a standard phase III study [21] embedded in the cRCT in Tanzania, the present paper reports a series of adapted phase II experimental hut (EHT) study conducted near the cRCT. The EHT study area had similar vector population and was designed to assess nets obtained from the main trial at regular intervals, which will enable to understand the impact of field conditions, wear-and-tear, and insecticidal deterioration on the bio-efficacy of the dual-AI LLINs on entomological outcomes [21]. This approach aimed to relate the study bio-efficacy outcomes to the epidemiological and entomological outcomes observed in the cRCT, while also establishing new standards of evaluation for these innovative products.

## Methodology

### Experimental hut study site

**Fig 1:**
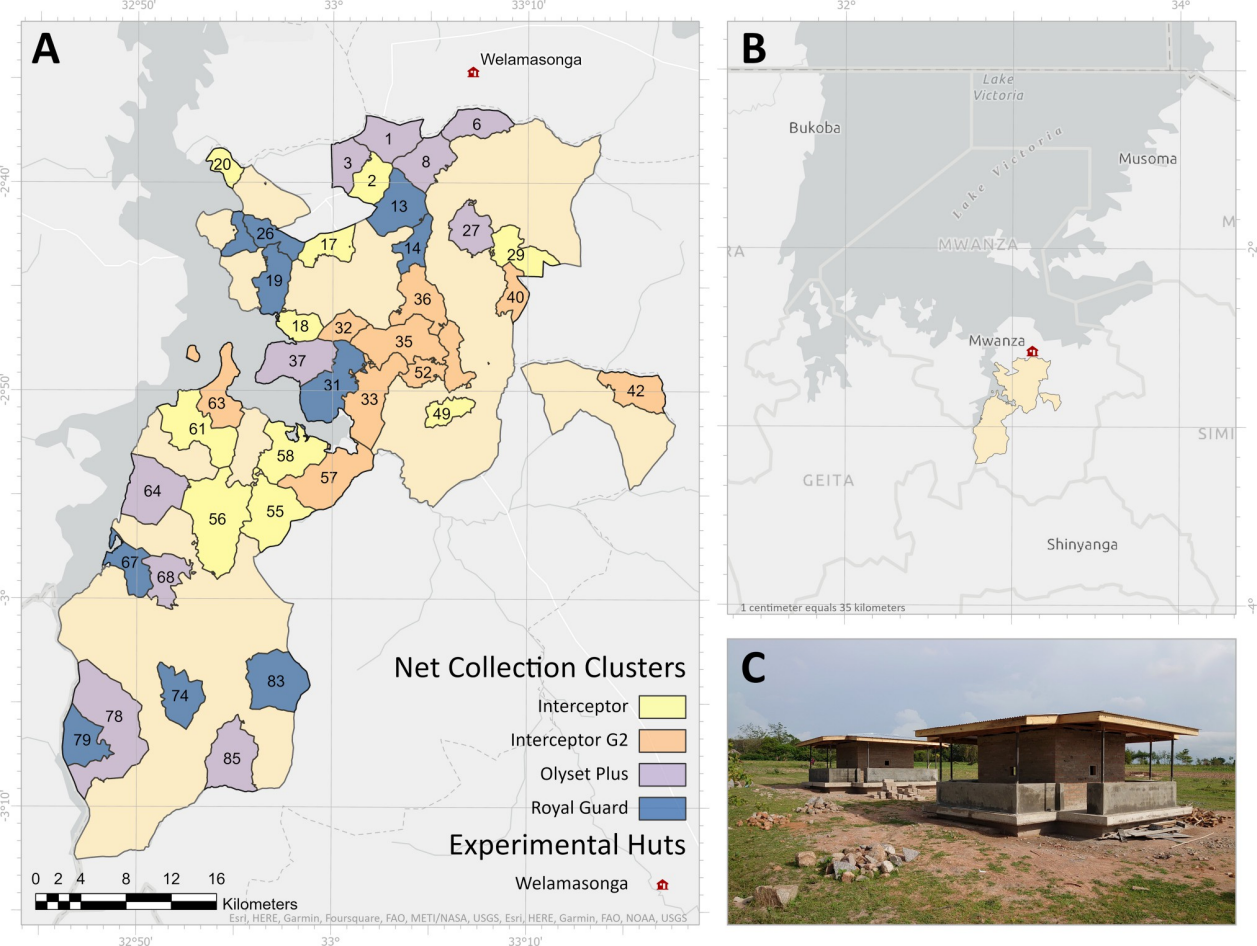
Map showing the location of experimental huts in the north part (B &C) of the main cRCT area and the clusters where the net was collected (A).The map was created with ArcGIS software and all geographical and administrative data were sourced from GADM: https://gadm.org/license.html.

The experimental hut (EH) study was conducted in Welamasonga village (2°34.673’ 33°07.170’), Magu district Mwanza, Tanzania, between 2020 to 2022. In this village, six east African experimental huts were constructed in the north part of the Misungwi cRCT area [21] (see Fig 1). A detailed description of the cRCT area can be found in the protocol published elsewhere [16].

Magu experiences the same climatic conditions as the cRCT site, characterized by two rainy seasons (March–May and October–December) separated by a long dry season (June–August or September) and a short dry season (December or January–February) [21]. The area surrounding the experimental huts consists of open fields used for cultivating rice and vegetables, with irrigation from ponds. The main vector species in this area are *An*. *funestus* sensu stricto (s.s), *An*. *arabiensis* and *An*. *gambiae* sensu stricto (s.s). The composition of these species varies with the season, with *An*. *gambiae* senso lato (s.l) dominating during the rainy season and *An*.*funestus* s.l. during the dry season.

Baseline resistance monitoring using CDC bottle assay reported a 69% mortality (24 hours post exposure) in *An*. *gambiae* s.l. exposed to the diagnostic dose of alpha-cypermethrin and 12% mortality for those exposed to permethrin. While a 25% mortality was reported for *An*. *funestus* s.l. after exposure to permethrin.

### Net distribution and its characteristics

The nets rotated in the experimental hut were collected from Misungwi district, Mwanza, Tanzania where the cRCT was conducted. The cRCT spanned between 2019 and 2022 and aimed to assess the effectiveness of two dual-AI LLINs and a pyrethroid-PBO LLIN, compared to standard pyrethroid-only nets against malaria infection [16]. In January 2019, study nets were distributed across the study arms in 84 clusters within the study area. Before distribution, six new nets from each brand were retained to be tested in the experimental huts. Characteristics of the four LLIN distributed were as followed: 1/ Royal Guard, a polyethylene net combining 225 mg/m^2^ of pyriproxyfen, which is known to disrupt female reproduction and fertility of eggs, and 261 mg/m^2^ pyrethroid alpha-cypermethrin; 2/ Interceptor G2, a polyester material incorporating two adulticides with differing modes of action; 200 mg/m^2^ of chlorfenapyr (a pyrrole) and a 100 mg/m^2^ of pyrethroid (alpha-cypermethrin;); 3/Olyset Plus, a polyethylene material which incorporates a synergist (400 mg/m^2^) PBO, to enhance the potency of pyrethroid insecticides and 800 mg/m^2^ of pyrethroid permethrin; 4/ Interceptor, a polyester filament coated with 200 mg/m^2^ of alpha-cypermethrin as a comparator to highlight the effect of the partner AI (Table 1).

**Table 1:** EHT treatments evaluated.

Treatment	Status of the net	Number of nets
1. Olyset Plus	Field collected	30 nets (tx*)
	New unwashed with 6 holes	6 nets (t0**)
2. Interceptor G2	Field collected	30 nets (tx)
	New unwashed with 6 holes	6 nets (t0)
3. Royal Guard	Field collected	30 nets (tx)
	New unwashed with 6 holes	6 nets (t0)
4. Interceptor	Field collected	30 nets (tx)
	New unwashed with 6 holes	6 nets (t0)
5. Positive control	New Interceptor with 6 holes	6 nets (t0)
6. Negative control	New untreated with 6 holes	6 nets (t0)

*tx are nets collected at 12, 24, and 36 months.

** new unwashed net.

### Sample size and sampling/withdrawal of LLIN for experimental hut trials

A total of six treatments were rotated during each EHT study. In addition to the four types of nets collected in the community, two other treatments were included, new standard LLIN (Interceptor as positive control) and untreated nets (negative control) were included [19] as shown in Table 1 below.

This study collected 30 LLINs per arm from the community at each time point. Since each EHT study was conducted over 36 nights and each intervention net was tested for one night, an additional six new LLINs of each type (Royal Guard, Interceptor G2, Olyset Plus, and Interceptor) were added to treatment 1 to 4 (Table 1) to complete the rotation and assess the efficacy of those different net types when they are new.

One month after distribution, 1950 nets in 5 clusters per treatment arm were labelled with a unique number. Thirty of these labelled nets were collected at intervals of 6, 12, 24, 30 and 36 months. Nets collected at 6 and 30 months were not included in the experimental hut rotation due to time constraint and limited number of huts. The sampling design is explained in more detail elsewhere [21]. Each collected net in the community was replaced by a new net of the same type. However, these replacement nets were not included in the study. Households remained part of the eligible cohort until no enrolled nets were available.

Number and size of holes in field collected nets were assessed to estimate the total hole area in each net brand, following WHO guideline [19]. The nets were categorized based on the total area: “good” if the area was less than 79 cm^2^, “damaged” hole area ranged from 80 to 789 cm^2^, and “torn” if the hole area was greater than 790 cm^2^ [22].

### Experimental hut study

The six experimental huts used for the study were built following an east African design but without veranda. Each EHT study was done over a 6-week period. Sleepers were rotated between huts on successive nights to account for individual attractiveness, and treatments were rotated every week following a random Latin square design. For each hut trial, during the 36 nights, each net (30 tx and 6 t0) from each treatment (Olyset Plus, Interceptor G2, Royal Guard and Interceptor) was rotated every night. For all new intact LLINs, six holes of 4 x 4 cm were cut following WHO guidelines [19].

During each night collection, cloth sheets were laid on the floor of each huts and sugar solution was provided at night in the window traps to reduce mosquito mortality [23]. The day after net installation in the hut, mosquitoes were collected using standard mouth aspirators from the room (floor, walls and ceilings), inside net, and window trap. The mosquitoes were packed in paper cups labelled with the collection date, hut number, net type, collection area (window/room/net) and collector initials. All mosquitoes were sent to the National Institute for Medical Research (NIMR) insectary. Mosquitoes were identified morphologically to species [24] and categorized by gonotrophic status (i.e., unfed, freshly fed, semi-gravid and gravid).

Mosquito mortality was monitored everyday up to 72 hours in a controlled environment. The effect of pyriproxyfen on reproductive outcomes was assessed on mosquitoes collected from the huts deployed with Royal Guard, Interceptor and untreated nets by dissecting gravid *Anopheles* 72-hours after collection when eggs should normally have fully matured [25, 26].

After a 72-hour holding period, *An*. *gambiae* s.l. were further identified to species-level using a TaqMan PCR assay to distinguish *An*. *gambiae* s.s. from *An*. *arabiensis* [27] and the same was done for *An*.*funestus* s.l. to distinguish between *An*.*funestus* s.s and *An*. *parensis* [28]. Half of the blood fed and unfed, alive and dead mosquitoes were packed in RNAlater for determination of species and cytochrome P450 expression levels using qPCR [29, 30].

Nets collected at each time point were assessed in the same year, with those collected after 12 months of use tested in 2020, those collected after 24 months in 2021, and those collected after 36 months in 2022. To account for vector seasonality and estimate net efficacy against different *Anopheles* species, four hut studies were conducted for nets collected at each time point over a year [21].

### Resistance monitoring

Wild *An*.*gambiae* s.l. and *An*.*funestus* s.l. were collected from houses adjacent to the experimental hut site around 6:00am to 7:00am in parallel of EHT study. Mosquitoes were morphologically identified to species and kept for three days to allow digestion of blood meal before bioassay testing. Resistance intensity to the insecticides contained in each LLIN was assessed using WHO/CDC bottle bioassays every year. Mosquitoes were exposed to the diagnostic doses of alpha-cypermethrin or permethrin and increased to 2, 5 and 10 times of diagnostic concentration for 30 minutes, for chlorfenapyr (100 μg/ml), pyriproxyfen (100 μg/ml) for 60 minutes; PBO pre-exposure was also performed using WHO tube bioassays followed by CDC exposure to pyrethroid.

### Outcome measures

The primary outcomes vary for each product and are on the mode of action of the active ingredient or synergist being evaluated following WHO recommendation [13, 19] as follows:-

Primary outcomes were 1/ 72-hour mortality for Interceptor G2, 2/24-hour mortality for Olyset Plus and 3/fertility for Royal Guard, calculated as the proportion of blood fed females alive at 72-hours with fully mature eggs. Secondary outcomes were: 1/ 24-hour mortality for Royal Guard and for all the nets 2/ blood feeding: proportion of blood fed mosquitoes collected, 3/deterrence: percentage reduction in density of *Anopheles* in treatment huts compared to negative control huts (fitted with untreated nets), 4/ exophily: proportion of mosquitoes that exited early and were found in window traps compared with the untreated control huts.

### Data analysis

Data analysis was performed using STATA software version 17. After data cleaning, four nights of collections were removed due to reporting errors. Descriptive statistics were used to estimate the proportion of mosquito species collected each year.

Mixed effect generalized linear models with logit link function were used to compare proportional outcomes (mortality, blood feeding and fecundity) of Interceptor G2, Royal Guard and Olyset Plus to reference Interceptor net (pyrethroid only field collected). Each models included treatment, hole index and net age as fixed effect and hut, sleeper and week as random effect to account for variation between sleepers, huts, collection weeks and seasonality. The interaction between treatment and net age was reported using odds ratios with their 95% confidence intervals (CI) and p-values were used to assess statistical significance at the 0.05 level for comparisons of mortality and blood-feeding. Mortality and blood feeding graphs were plotted using ggplot in R software.

For resistance data, lethal dose values (LD25, LD50, LD95 and LD99) were calculated using a probit model with log-10 transformed data in IBM SPSS v28 software. The curve estimation was based on the probability of mosquito death as a function of the total number of mosquitoes and insecticide dose [31]. Point estimates of LDs and 95% CIs were then back-transformed to their original scale to obtain the reported values, indicating the difference in diagnostic dose of an insecticide required to kill 25%, 50%, 95% or 99% of tested mosquitoes. Comparisons of LD50 values among clusters and/or years were statistically estimated using the Relative Median Potency (RMP), calculated as the ratio of point estimates with simultaneous 95% CIs. Comparisons of “potency” in this context are median lethal concentrations/doses. A ratio of “1” is considered insignificant, meaning LD50 was equal among comparison groups. Reduction in fertility was calculated as ((proportion fertile in control -proportion fertile in treatment)/ proportion fertile in untreated net control)*100. P-value less than 0.05 was considered as significant.

## Results

### Species composition and outcomes in negative and positive control huts over the 3 years

A total of 12 experimental hut trials were conducted between 2020 and 2022, resulting in 2,588 collection nights. During this period, 17,040 male and female mosquitoes were collected with 87% (14,841/17,040) of them being female. Among the female mosquitos, 26% (3,925/14,841) were identified as *Anopheles*, while the remaining were *Culex quinquefasciatus*.

For all collection years combined, 63.4% (2488/3925) of the *Anopheles* were identified as *An*. *gambiae* s.l. while the remaining 36.6% (1437/3925) were *An*. *funestus* s.l.. Among the *An*. *gambiae* complex identified to species-level, 56.8% (673/1185) were determined to be *An*. *gambiae* s.s. while the rest were classified as *An*. *arabiensis*. Notably, the proportion of *An*. *gambiae* s.s. was highest in 2020 and subsequently decreased over the years (as shown in Table 2).

**Table 2:** Hut trials entomological characteristics (density and species composition) over the three years of follow-up.

	Year 1: 2020		Year 2:2021		Year 3:2022	
Total hut/night collection	863		864		864	
Total mosquitoes collected (female)	4320 (3869)		7424 (6612)		5296 (4360)	
Total *Anopheles* collected (female)	1974 (1611)		1904 (1457)		1204 (857)	
*Anopheles* vectors: N, mean [95%CI]	1611	1.9 [1.7–2.0]	1457	1.7 [1.5–1.8]	857	0.9 [0.9–1.1]
*Culex* species: N, mean [95%CI]	2270	2.6 [2.4–2.8]	5155	5.9 [5.2–6.7]	3503	4.1 [3.7–4.4]
**Species composition n/N, % [95%CI]**						
*An*. *gambiae*s.l./*Anopheles*	1161/1611, 72.1%, [68.9–75.1]		842/1457, 57.8% [51.9–63.4]		485/857, 56.6% [50.4–63.5]	
*An*. *funestus*s.l./*Anopheles*	450/1611, 27.9% [24.4–30.7]		615/1457, 42.2% [36.6–48.1]		372/857, 43.4% [36–49.6]	
*An*. *gambiae*s.s/*An*.*gambiae*s.l	347/410, 85.0% [80.7–87.8]		181/334, 54.0% [48.8–59.4]		143/435, 33.0% [29–37]	
*An*. *funestus*s.s/*An*.*funestus*s.l	151/153, 99.0% [94.8–99.6]		436/460, 95.0% [92.3–96.5]		321/368, 87.0% [83–90]	

*Anopheles* mortality for untreated nets (negative control huts) was less than 5% after 24-hours which meets the WHO recommended threshold, while the exophilic rate ranged between 62% and 68% depending on the year. Blood feeding was between 18% and 21% in huts fitted with a new standard pyrethroid LLIN Interceptor (positive control huts); 24-hour mortality was low and ranged from 8% to 12%. The mortality against *An*. *funestus* complex for the whole period of study varied between 4% to 10% while it ranged between 7% to 18% for *An*. *gambiae* complex (see S1 Fig).

### EHT results on mortality and blood-feeding

**Fig 2:**
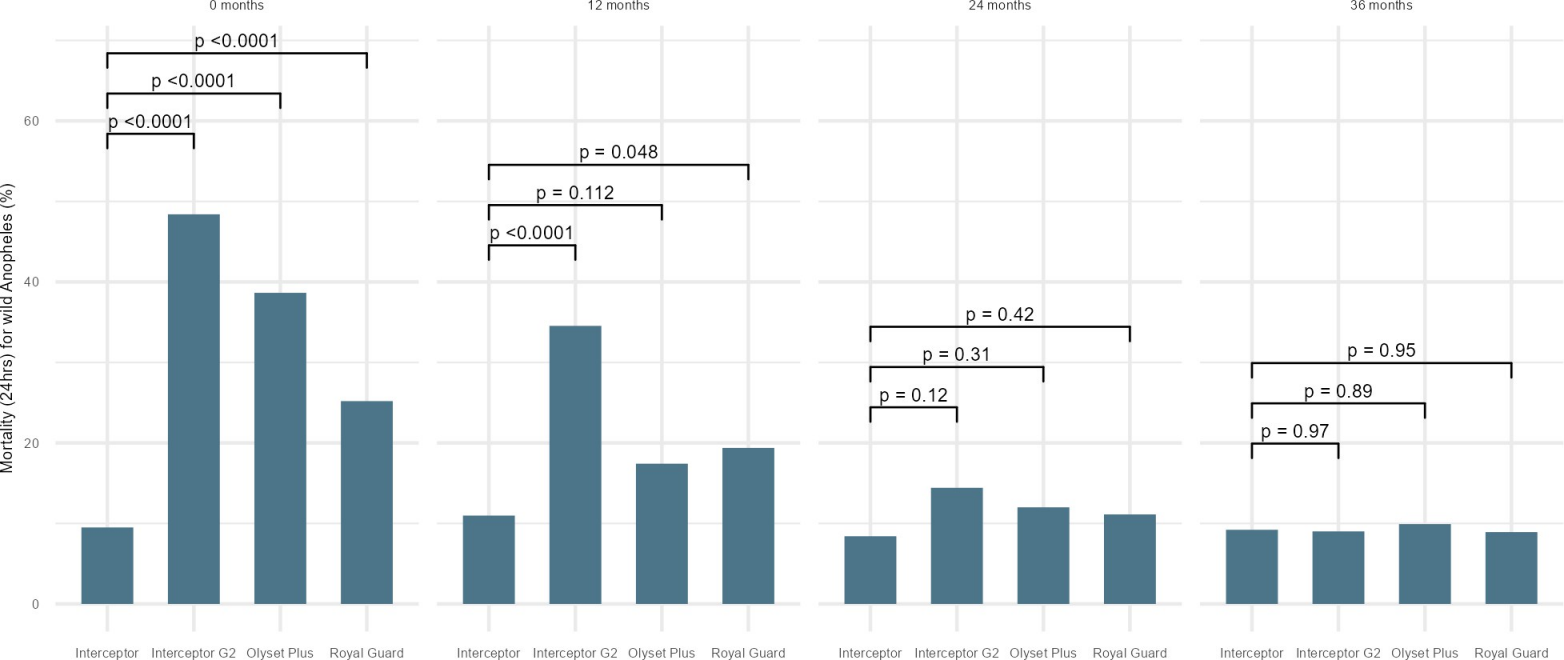
Model output mortality (24-hrs) of wild free flying female *Anopheles* in experimental hut by net type and age.

**Fig 3:**
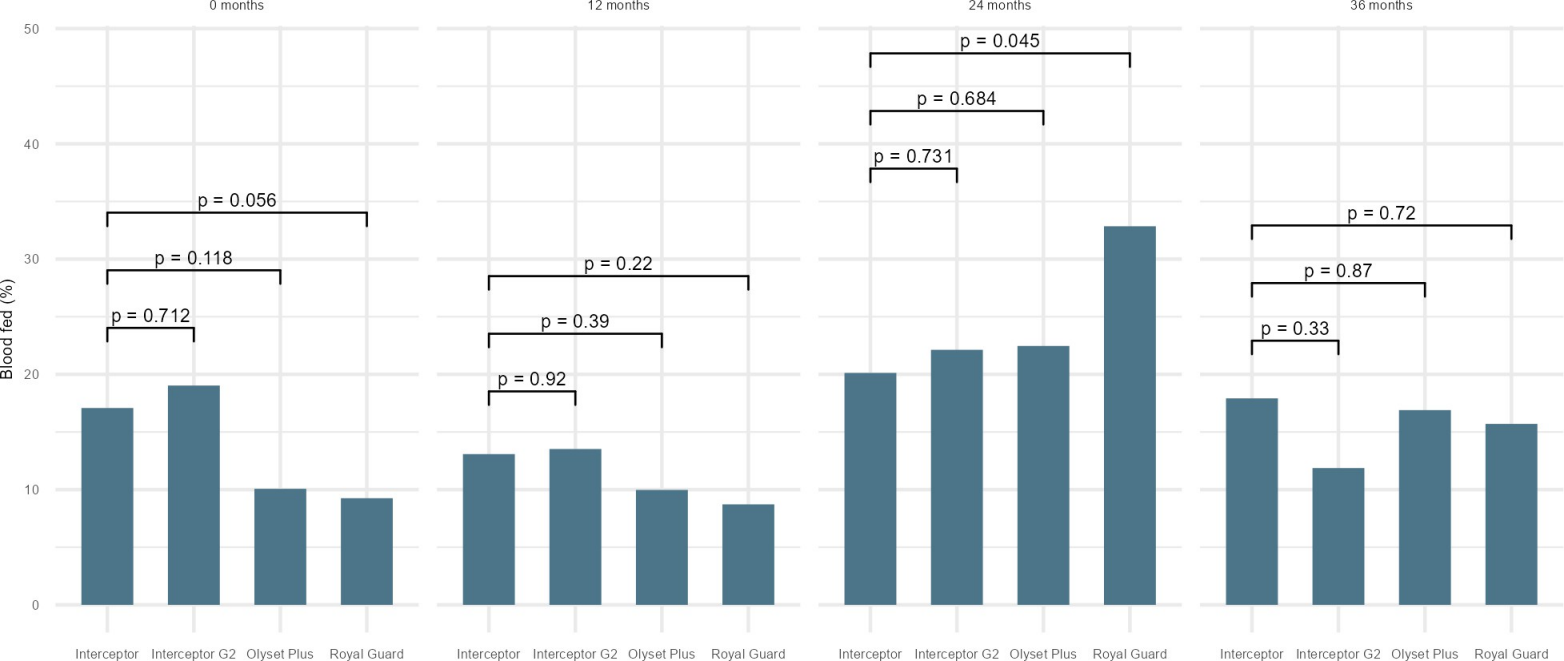
Model output blood feeding in wild free flying female *Anopheles* in experimental hut by net type and age.

There was higher 24-hour mortality in *Anopheles* collected in huts fitted with new (0 month) Olyset Plus compared to a new standard pyrethroid LLIN (raw data 38% vs 6%), adjusted odds ratio (OR): 13.6, 95%CI: 4.4–41.3, p-value<0.001 (Fig 2). Mortality in Olyset Plus dropped over time and while it remained slightly higher compared to standard pyrethroid LLINs of the same age, the difference was not significant after nets were used for 12 months or more (12 months: 17% vs 13%; OR: 2.1, 95%CI: 1.0–4.3; p = 0.112, 24 months: 12% vs 11%, OR: 1.4, 95%CI: 0.6–3.3; p = 0.310 and 36 months: 10% vs 7%, OR: 1.0, 95%CI: 0.3–3.5; p = 0.890) (see Fig 2 and S1 Table). By species, 24-hour mortality remained significantly higher in Olyset Plus compared to the standard pyrethroid LLIN in *An*. *gambiae* s.l. (23% vs 13%, OR: 2.6, 95%CI: 1.0–6.4; p = 0.045) at 12 months but no effect was observed on the *An*. *funestus* complex at this time point (see S2 Table 1B). Blood feeding for both species was lower in Olyset Plus compared to Interceptor LLINs up to 12 months but the difference was not significant at any of the following time points (see Fig 3).

**Fig 4:**
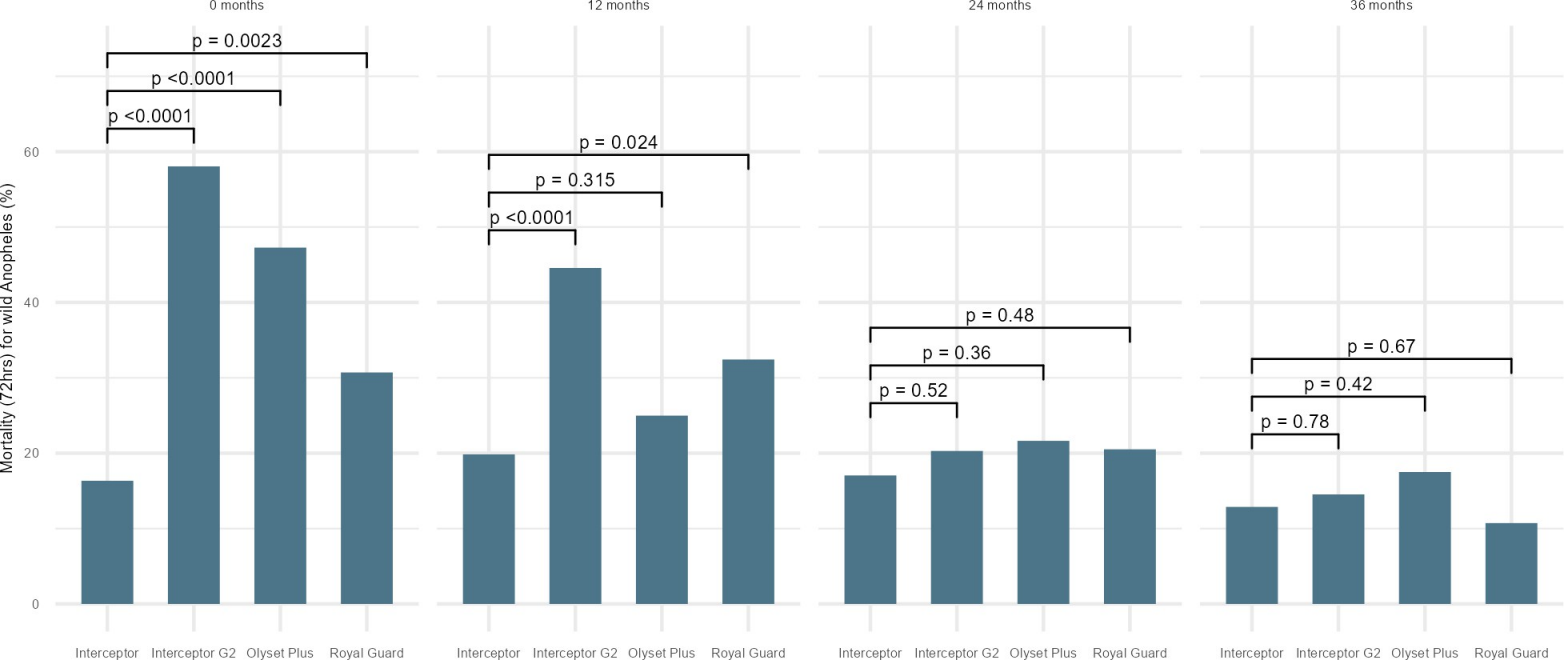
Model output mortality (72-hrs) of wild free flying female *Anopheles* in experimental hut by net type and age.

Interceptor G2 LLINs provided higher 72-hour mortality compared to Interceptor LLINs when new (raw data 58% vs 14%), (OR: 11.9, 95%CI: 4.8–29.7 p<0.001) and after 12 months of use (43% vs 21%, OR: 3.5, 95%CI: 1.9–6.6, p<0.001) (see Fig 4 and S1 Table). The effect was observed for both *An*. *gambiae* s.l. and *An*. *funestus* (see S2 and S3 Tables). At 24 and 36 months, the difference in mortality was no longer significantly higher. Similar findings were observed for 24-hour mortality (see Fig 2). In terms of blood feeding inhibition, there were no differences between Interceptor G2 compared to Interceptor LLINs at any time point (see Fig 3).

For Royal Guard, the primary outcome was effect on fertility. In Royal Guard LLINs huts, a total of 707 female *Anopheles* were collected, of which 133 were blood fed and only 89 alive after 72 hours and available for dissection (see Table 3). The fertility rate was 67% when Royal Guard was new and increased to 75% after 12 months of use. Meanwhile, it varied between 94% and 100% for *Anopheles* collected from untreated nets and standard pyrethroid Interceptor LLINs. At 24 and 36 months, all mosquitoes were found with fully mature eggs in Royal Guard and categorized as fertile. Interestingly, despite small numbers being dissected, a reduction in fertility was only observed for *An*. *gambiae* s.l. and not *An*. *funestus* s.l.. Mortality outcomes were also monitored and were found significant at 0 month and 12 months compared to Interceptor LLINs (S1 Table). Blood feeding was lower at 0, 12 and 36 months in Royal Guard compared to standard pyrethroid LLINs but the difference was not significant and decreased with time, while at 24 months more blood fed *Anopheles* were found in the Royal Guard huts (as shown in Fig 3 and Table 3) than in standard pyrethroid LLINs.

**Table 3:** Fertility in wild free flying female *Anopheles* in experimental hut fitted with Interceptor, untreated net and Royal Guard by age.

Net type	Total collected	Total BF*	Total alive 72hrs post collection	Total BF* alive 72hrs post collection	Number dissected	Total fertile	Percentage (%)	95CI
0months								
Untreated	104	32	100	32	26	26	100	-
Interceptor	113	23	98	23	16	15	94	62–99
Royal Guard	139	15	96	14	6	6	67	36–99
12 months								
Untreated	230	73	213	72	57	56	98	89–100
Interceptor	181	28	143	27	17	17	100	-
Royal Guard	222	24	152	23	12	9	75	41–93
24 months								
Untreated	156	57	150	57	37	37	100	-
Interceptor	170	38	136	37	30	30	100	-
Royal Guard	225	79	174	76	51	51	100	-
36 months								
Untreated	133	34	127	34	28	28	100	-
Interceptor	91	17	78	17	17	17	100	-
Royal Guard	121	20	107	20	20	20	100	-

*Blood fed

### EHT results on deterrence, exiting and net penetration

There was no clear pattern with deterrence for *An*. *gambiae* s.s.. For nets aged 0 and 24 months there was no deterrence effect in the treatment huts relative to huts fitted with untreated nets while at 12 and 36 months there was some reduction of *An*. *gambiae* s.s. entered in huts with treated nets (S4 Table). For *An*. *funestus* complex, regardless of net age there was some hut entry reduction in huts fitted with Interceptor G2 and standard pyrethroid Interceptor LLINs while that was not observed at any time point for Olyset Plus and Royal Guard (except at 36 months for the latter). Overall, the highest exit rate for *Anopheles* was found in treatment huts compared to untreated huts, but there were no significant differences between Interceptor G2, Royal Guard or Olyset Plus compared to Interceptor of the same age. Overall, penetration of nets by *Anopheles* was consistently lower for all the insecticide treated nets compared to untreated nets (S4 Table).

### Hole characteristics of nets sampled from the community and effect of hole and net age on vector blood feeding

The mean hole area for Interceptor G2 and Olyset Plus LLINs aged 12 months for the good category was 26.5cm^2^ and 27.9 cm^2^ respectively, while the mean hole area in Royal Guard was 16.2 cm^2^. On average, hole size increased with net age (see S5 Table). Overall, mortality and blood feeding observed was not impacted by the size or number of holes in any of the nets.

### Insecticide resistance in malaria vectors

Longitudinal changes in vector insecticide resistance intensity were assessed to determine their influence on vector mortality in the EHTs. Permethrin resistance intensity was high in *An*. *funestus* in year one LD50 = 292.9 [52.7–3906.3] but diminished over time (year two LD50 = 33.7 [20.5–81.7] and year three LD50 = 19.1 [13.5–36.2]). By comparison, levels of alpha-cypermethrin resistance intensity in *An*. *funestus* increased significantly during the EHTs (year two LD50 = 22.5 [12.9–65.1] and year three LD50 = 181.6 [61.8–1499.9]). In *An*. *gambiae* s.l., a similar decline in permethrin resistance intensity was evident (year one LD50 = 3417 [4.0–7319224.4], year two LD50 = 24.1 [15.5–48.3] and year three LD50 = 43.6 [26.4–100.0]). However, there was no parallel increase in alpha-cypermethrin resistance (year one LD50 = 0.24 [0.0–0.6], year two LD50 = 0.52 [0.2–0.9] and year three LD50 = 0.79 [0.3–1.3]). In both vector species complex, there was limited change in permethrin resistance intensity following PBO pre-exposure.

Following chlorfenapyr exposure, high 72-hour mortality was evident in both species complexes at year one (92% [95% CI: 87–97] and 91% [95% CI: 87–96] for *An*. *gambiae* s.l. and *An*. *funestus* s.l., respectively); complete (100%) mortality was observed for both *An*. *gambiae* s.l. and *An*. *funestus* s.l, in year two and three.

During pyriproxyfen testing, there was no impact on fertility in *An*. *funestus* s.l. in year one while an 8.1% [8/98] reduction was observed in *An*. *gambiae* s.l. in year one. For year two and three, only *An*. *gambiae* s.l. were tested and 8.8% [5/57] and 6.7% [1/15] reduction in fertility was observed respectively. Overall, sterility effect was less than 10%.

**Fig 5:**
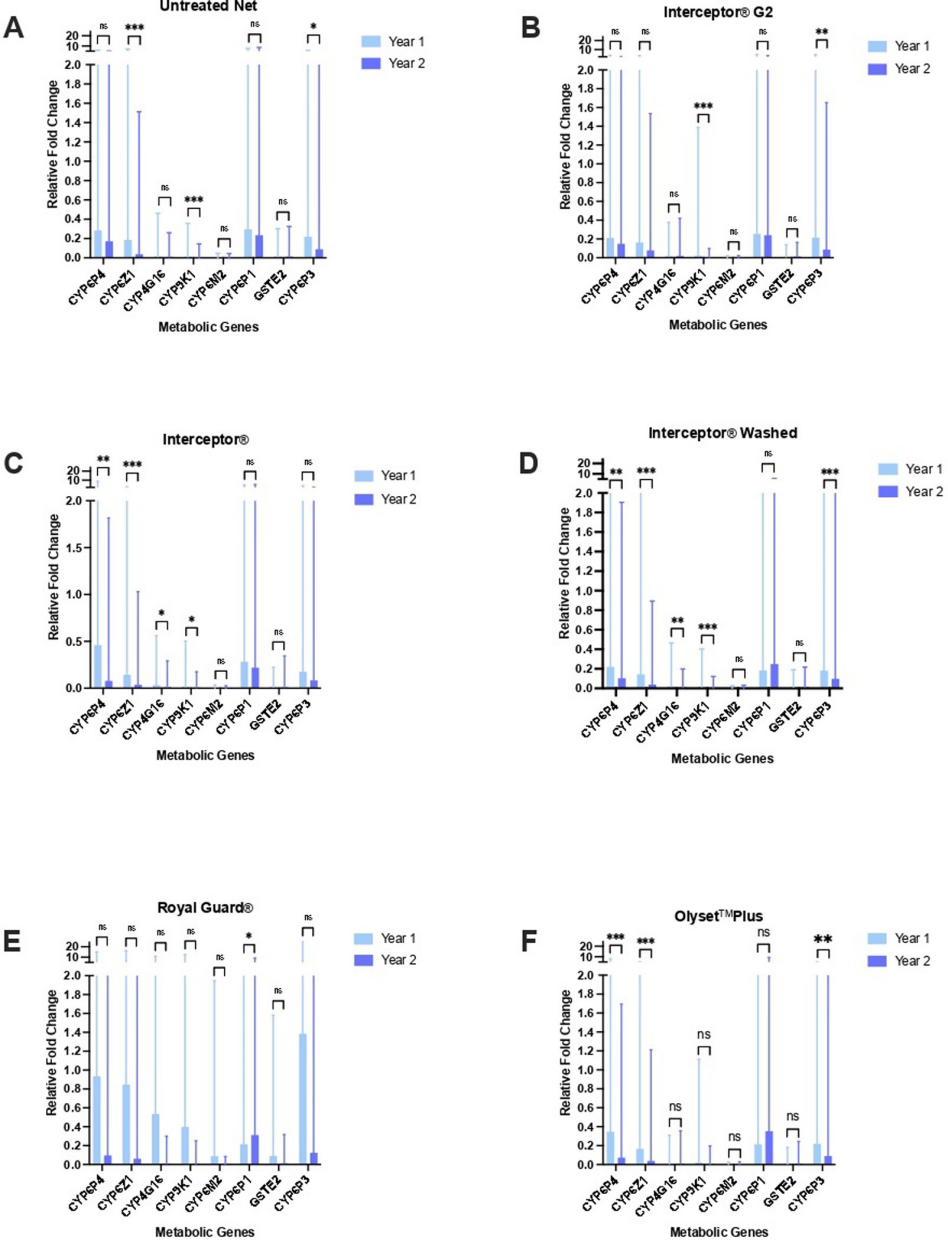
Gene expression in wild field collected *An*. *gambiae* s.l. relative to colony susceptible population over two years in untreated net (A), Interceptor G2 (B), standard Interceptor (field collected) (C), standard Interceptor (washed once) (D), Royal Guard(E) and Olyset Plus (F).

Dynamic changes in expression of eight metabolic genes (CYP6P4, CYP6Z1, CYP4G16, CYP9K1, CYP6M1, CYP6P1, CYP6P3 and GSTF2) were observed in PCR-confirmed *An*. *gambiae* s.s. between trial years one and two (Fig 5). CYP9K1, CYP6M2 and GSTE2 displayed consistent minimal over-expression relative to the susceptible colony strain. By comparison to the untreated net arm, significant declines in CYP6P4 expression were observed in Interceptor (washed and unwashed) and Olyset Plus exposed mosquitoes and no significant decrease in CYP6Z1 expression was present in Royal Guard and Interceptor G2 exposed mosquitoes between years one and two; while a significant increase in CYP6P1 expression was apparent in vectors collected from Royal Guard EHs. In Fig 5 error bars represent 95% confidence intervals. Statistically significant differences in expression levels between trial years are indicated as follows: ns = not significant; * = p-value <0.05; ** = p-value <0.01; *** = p-value< 0.001.

## Discussion

These series of experimental hut trials were conducted to assess the impact of interventions (dual-AI LLINs) on mortality and blood-feeding rates in resistant *An*. *gambiae* s.l. and *An*. *funestus* s.l. vector populations and to better understand the associated cRCT outcomes. When new, Interceptor G2 and Olyset Plus provided a significantly higher killing effect compared to the reference net (Interceptor) against wild-resistant *Anopheles* mosquitoes found in Magu district. Mortality was also generally higher for these two nets at 12 months, but there was only strong evidence for a difference for Interceptor G2. Royal Guard did not have a significant impact on fertility outcomes at any of the time points.

The highest effect of Interceptor G2 on vector mortality was observed for both vectors *An*. *gambiae* s.l. and *An*. *funestus* up to 12 months, with no observable difference after 24 and 36 months of net use compared to Interceptor. Previous experimental hut trials done in Tanzania [32] assessing new unwashed Interceptor G2 reported a 72-hour mortality around 50% against *An*. *funestus* similar to our findings, while mortality was in general higher in Benin (71%) and in Cote d’Ivoire (87%) against *An*. *gambiae* s.l.. Interestingly, in these EHTs, after 20 standardised washes (supposed to mimic a 36 month field net) 72-hour mortality was 52% in Tanzania, 65% in Benin [15] and 82% in Cote d’Ivoire [33], all much higher than what we found in our EHT at any given time point. In our study, the insecticidal content over time depleted quicker compared to those exposed to laboratory washing procedures; indeed, the residual concentration of chlorfenapyr was only 8% of the initial content after 36 months of operational use while the chlorfenapyr retention observed in nets washed 20 times were 32% in Tanzania [32] and 37% in Benin [15, 32].

Overall mortality induced by field collected Olyset Plus LLINs was significantly higher than pyrethroid-only net (Interceptor) when the nets were new but at no other time point. When looking at the effect per species mortality was also significantly higher in *An*. *gambiae* s.l. at 12 months which was not the case for *An*. *funestus*. In the cRCT conducted as part of this study [16], Olyset Plus LLIN was only effective for a year while a previous cRCT conducted in Muleba-Tanzania showed that Olyset Plus LLIN arm had lower malaria than Olyset Net [12] and provided personal protection [34] up to 21 months of use in an operational setting. The EHT results from the present study suggest that Olyset Plus might control *An*. *gambiae* s.s. better than *An*. *funestus*, which could explain the difference between those two cRCTs as in Misungwi the main vector was *An*. *funestus* [35] while in Muleba it was *An*. *gambiae* s.s. [12]. The effectiveness of Olyset Plus declined as the net aged, which aligns with the observed reduction in both permethrin and PBO content after 36 months of community use (8.3 g/kg vs 20 g/kg and ~0.7 g/kg vs 10 g/kg, respectively, when new). Similar findings were reported in Uganda [36] where there was a low retention (3.7 g/kg) of PBO content 25 months after the distribution of Olyset Plus LLINs. In Kenya [37], 87% of PBO and 52% of permethrin were lost after 36 months of community use. Notably, PBO retention was higher when nets were subjected to 20 laboratory washes (2.0 g/kg) in Benin [38].

In the case of Royal Guard, no significant sterility effect was evident over the three-year period. Only 33.3% of *Anopheles* were deemed sterile after exposure to new, unwashed Royal Guard nets, and this figure decreased to 25% after one year of use. There was no observed sterility effect at 24 and 36 months. Notably, the impact of Royal Guard on mortality was noticeable only up to 12 months. The limited number of blood-fed *Anopheles* still alive at 72-hours suggests that the combination of pyrethroid and pyriproxyfen in the first year may have had a greater impact on mortality and blood feeding than on fertility. A study in Benin [14] using Royal Guard LLINs indicated a reduction in the reproductive ability of mosquitoes up to 20 washes in a laboratory setting. However, the effect was lower (25%) in experimental huts. Another pyriproxyfen net brand, Olyset Duo, evaluated in Moshi, Tanzania, against *An*. *gambiae* s.l. reported 34.6% fertility after exposure in tunnel tests following 20 washes [39]. Nonetheless, an entomological assessment of this brand in a cRCT in Burkina Faso revealed that the sterility effect was only observed for one month after LLIN distribution [40]. The difference in performance between laboratory and community studies could be explained by more stringent washing methods and abrasion during daily use. In our study only 28% of pyriproxyfen and 62% of alpha-cypermethrin remained on the nets after 36 months while insecticide retention was higher when washed in the laboratory (57% and 76% for pyriproxyfen and alpha-cypermethrin, respectively) [14].

It is important to note that all these studies were carried out using unwashed nets, with the nets washed 20 times as a proxy for a 3-year net used in the community. It is worth considering that insecticide retention on nets in community may be lower than in LLINs washed 20 times, as community washing process can be more intense and the nets more subject to friction during general use [17]. In addition, hole size in aged nets in community may differ from the 4x4 cm standardized holes created in washed nets. Data from experimental hut trials conducted with ‘real-life’ field nets can help explain why Royal Guard LLINs had a more moderate effect on mosquito density and transmission in community trials, and why Olyset Plus LLINs did not last for more than 24 months in recent trials [16, 41].

To prequalified new LLIN products, WHO recommends different phases of evaluation including phase II laboratory wash resistance which is assumed to corelate with 36 months of community use [19]. However, this study reveals that wash resistance (20 times) does not correlate with the 36 months LLIN which may be due to differences in washing methods and quality of the nets. Furthermore; all the previous studies (wash resistance studies) were done on small batch of nets while this study was carried in the larger cRCT. The WHO could review guidelines for evaluation (phases I, II and III) and increase number of washes and recommend a more abrasive washing method to mimic 36 months LLIN in a field setting. Others have speculated that the quality of nets has reduced over the years [42]. This could also be the case in this study; all previous phase II studies were conducted on a small batch of nets made specifically for the study’s purpose, whereas here, a subsample taken from 45,000 nets distributed was used. Additional standard EHT with unwashed and 20-wash nets could be conducted using nets purchased in bulk for distribution by the malaria control program to verify this assumption.

Resistance intensity monitoring demonstrated high resistance to permethrin in *An*. *funestus* in the first year, which was lost in the second and third year. By comparison, alpha-cypermethrin resistance intensity increased significantly over trial years in *An*. *funestus*, which aligns with resistance monitoring results from the main cRCT [31]. This may be due to an increase in the proportion of zoophilic *An*. *arabiensis* recorded resting in the huts [31].

Chlorfenapyr demonstrated high killing effect in all three years of resistance monitoring without evident selection to this AI, while pyriproxyfen failed to induce significant sterility effect against *An*. *gambiae* s.l. and *An*. *funestus* complex throughout the same period. Pyriproxyfen resistance results is consistent with those observed in cRCT study site and could explain the relatively small effect observed in both cRCT and EHT presented [31]. The molecular analysis for the detoxification enzymes revealed over-expression of genes (CYP6P3) in Interceptor, Interceptor G2 and Olyset Plus which strongly metabolize pyrethroid insecticide, as it was observed in the study examining the functional genetic keys that confer resistance in African malaria vectors, *An*. *gambiae* s.l. [43–45].

One of the limitations in this study was the variation in species composition over time which could explain why some of the nets did not perform as anticipated. Future studies may evaluate nets of different ages side-by-side to control for this heterogeneity across years. A second limitation is that a relatively small number of blood fed *Anopheles* alive at 72-hours were available for dissection and therefore the impact of Royal Guardon sterility might have been slightly underestimated. Third limitation is that, the study only used 30 nets sampled from five clusters per arm out of the 21 clusters so they are probably not representative of the overall community in Misungwi.

Overall, the reduction in efficacy of Olyset Plus and Royal Guard LLINs observed in the EHTs seems to match the entomological and epidemiological findings in the cRCT conducted in Misungwi. Indeed, Olyset Plus provided superior protection against malaria and vectors outcomes compared to Interceptor up to 12 months only [16] while there was no significant differences for Royal Guard. However this was less clear for Interceptor G2. While in the current EHTs Interceptor G2 outperformed Interceptor only up to 12 months, the cRCTs outcomes reported significant malaria prevalence reduction and vector densities in the Interceptor G2 arm compared to standard LLIN at 24 months [16, 17] and even up to 36 months [41]. Differences in species composition in the EHT and cRCTs area could explain the difference as there was a majority of *An*. *funestus* found in the cRCTs while *An*. *arabiensis* was the predominant species in the EHT when the 24 and 36 months nets were tested. In the cRCT, it was reported that *An*. *arabiensis* was not well controlled by any of the dual active ingredient LLINs, including Interceptor G2, due to its exophilic behaviors [35]. Given that chlorfenapyr is a multifaceted insecticide, differences between cRCT and EHT outcomes may be explained by potential effects of chlorfenapyr on parasite development in mosquitoes [46, 47]. This could explain the stronger and longer lasting effect observed in the community cRCT which may not be captured in EHT. What the result of the present EHT also highlighted is that the reduction in effect observed in the cRCT in Tanzania over the three years might not only be due to reduction in net usage but also to the sharp decrease in partner AI or PBO resulting in lower killing effect as the net aged.

Modelling of EHT data have been used to parameterize mechanistic models for malaria vectors and predict the epidemiological efficacy of LLINs [48]. The use of EHT data which better correlates with cRCT outcomes as observed in the present study may improve the fit of these models and could be sufficient for the evaluation of second in class products.

## Conclusion

Olyset Plus LLINs exhibited higher mortality rates, and Royal Guard LLINs demonstrated greater sterility effect compared to Interceptor LLINs, but only when newly introduced. These findings align with the results of the cluster-randomized controlled trial (cRCT), which reported a time-limited effect (up to 12 months) on epidemiological and entomological outcomes. In contrast, Interceptor G2 LLINs exhibited superiority against *An*. *gambiae* s.l. and the *An*. *funestus* complex compared to Interceptor LLINs, but this advantage was observed only for up to 12 months. Interestingly, Interceptor G2 LLINs provided extended protection against malaria for a period of 3 years in associated cRCTs. Further investigation is needed to explore additional effects, such as the effect of chlorfenapyr on parasite reduction, to fully comprehend its impact on malaria transmission. Additionally, conducting standard and adapted EHT in various contexts will help confirm the residual efficacy of the dual active ingredient LLINs and support the development of longer-lasting nets.

## Supporting information

S1 Fig Control mortality for *An*. *Gambiae* s.l and *An*.*funestus* complex collected from experimental hut trial. (DOCX)

S1 Data Stata file for the experimental hut data. (DTA)

S2 Data Excel file for Gene expression in wild field collected *An*. *gambiae* s.l. relative to colony susceptible population over two years in untreated net. (XLSX)

S1 Table Percent mortality and blood feeding with their odds ratio and 95%CI. (DOCX)

S2 Table Percent mortality and blood feeding for *An*. *gambiae* s.l with their odds ratio and 95%CI. (DOCX)

S3 Table Percent mortality and blood feeding for *An*. *gambiae* s.l with their odds ratio and 95%CI. (DOCX)

S4 Table Total collected mosquitoes per treatment per time point inside net and exit traps with percent deterrence. (DOCX)

S5 Table Effect of hole on blood feeding in *Anopheles* mosquitoes collected. (DOCX)
