# Supplementary material for: Bio-efficacy of field aged novel class of long-lasting insecticidal nets, against pyrethroid-resistant malaria vectors in Tanzania: A series of experimental hut trials
Source: PLOS Glob Public Health. 2024 Oct 4;4(10):e0002586. doi: 10.1371/journal.pgph.0002586 (PMC11451999; doi:10.1371/journal.pgph.0002586)
Supplement: S1 Fig — (DOCX) [file pgph.0002586.s001.docx]

S1 Fig: Control mortality for *An. Gambiae* s.l and *An.funestus* complex collected from experimental hut trial.
