# Supplementary material for: Bio-efficacy of field aged novel class of long-lasting insecticidal nets, against pyrethroid-resistant malaria vectors in Tanzania: A series of experimental hut trials
Source: PLOS Glob Public Health. 2024 Oct 4;4(10):e0002586. doi: 10.1371/journal.pgph.0002586 (PMC11451999; doi:10.1371/journal.pgph.0002586)
Supplement: S1 Table — (DOCX) [file pgph.0002586.s004.docx]

S1 Table: Percent mortality and blood feeding with their odds ratio and 95%CI.

|  |  | 24 hours mortality % (n/N) | | | | 72 hours mortality % (n/N) | | | | BF % (n/N) | | | | |
| --- | --- | --- | --- | --- | --- | --- | --- | --- | --- | --- | --- | --- | --- | --- |
|  | Total collection | mortality % (n/N) | OR* | 95%CI | p value | mortality % (n/N) | OR* | 95%CI | p value | BF (n/N) | OR* | 95%CI | p value |  |
| **0 month** |  |  |  |  |  |  |  |  |  |  |  |  |  |  |
| Interceptor (reference) | 72 | 6% [7/111] | 1 |  |  | 14% [ 15/110] | 1 |  |  | 21% [23/111] | 1 |  |  |  |
| Interceptor G2 | 72 | 48% [49/102] | 19.9 | 6.5 - 61.6 | <0.001 | 58% [59/102] | 11.9 | 4.8 - 29.7 | <0.001 | 28% [29/102] | 1.1 | 0.4 - 2.7 | 0.712 |  |
| Royal Guard | 72 | 25% [ 35/139] | 7.0 | 2.3 - 20.9 | <0.001 | 31% [43/139] | 3.7 | 1.5 - 8.7 | 0.0023 | 9% [13/139] | 0.5 | 0.2 - 1.2 | 0.056 |  |
| Olyset Plus | 72 | 38% [43/112] | 13.6 | 4.4 - 41.3 | <0.001 | 46% [52/112] | 7.0 | 2.8 - 17.1 | <0.001 | 10% [11/112] | 0.5 | 0.2 - 1.2 | 0.118 |  |
| **12 month** |  |  |  |  |  |  |  |  |  |  |  |  |  |  |
| Interceptor (reference) | 120 | 13% [23/181] | 1 |  |  | 21% [38/181] | 1 |  |  | 15% [28/181] | 1 |  |  |  |
| Interceptor G2 | 120 | 35% [68/193] | 4.6 | 2.3 - 9.5 | <0.001 | 44% [ 84/193] | 3.5 | 1.9 - 6.6 | <0.001 | 18% [34/193] | 0.9 | 0.4 - 1.9 | 0.92 |  |
| Royal Guard | 120 | 19% [43/222] | 2.1 | 1.0 - 4.4 | 0.048 | 33% [73/222] | 2.1 | 1.1 - 3.8 | 0.315 | 8% [18/222] | 0.6 | 0.3 - 1.3 | 0.22 |  |
| Olyset Plus | 119 | 17% [44/252] | 2.1 | 1.0 - 4.3 | 0.112 | 27% [67/252] | 1.5 | 0.8 - 2.9 | 0.212 | 10% [ 32/252] | 0.6 | 0.3 - 1.4 | 0.39 |  |
| **24 month** |  |  |  |  |  |  |  |  |  |  |  |  |  |  |
| Interceptor (reference) | 120 | 11% [ 18/170] | 1 |  |  | 20% [34/170] | 1 |  |  | 22% [ 38/170] | 1 |  |  |  |
| Interceptor G2 | 120 | 15% [27/180] | 1.9 | 0.8 - 4.2 | 0.12 | 20% [36/180] | 1.3 | 0.7 - 2.6 | 0.52 | 24% [ 43/180] | 1.1 | 0.5 - 2.3 | 0.731 |  |
| Royal Guard | 120 | 11% [ 25/225] | 1.3 | 0.6 - 3.1 | 0.42 | 20% [46/225] | 1.1 | 0.6 - 2.2 | 0.48 | 36% [80/225] | 1.8 | 0.9 - 3.6 | 0.045 |  |
| Olyset Plus | 120 | 12% [25/208] | 1.4 | 0.6 - 3.3 | 0.31 | 24% [49/208] | 1.2 | 0.6 - 2.3 | 0.663 | 25% [52/208] | 1.1 | 0.5 - 2.2 | 0.684 |  |
| **36 month** |  |  |  |  |  |  |  |  |  |  |  |  |  |  |
| Interceptor (reference) | 120 | 7% [ 6/91] | 1 |  |  | 14% [13/91] | 1 |  |  | 19% [17/91] | 1 |  |  |  |
| Interceptor G2 | 120 | 9% [9/95] | 1.6 | 0.4 - 5.7 | 0.97 | 15% [14/95] | 1.5 | 0.6 - 4.1 | 0.78 | 13% [12/95] | 0.6 | 0.2 - 1.7 | 0.33 |  |
| Royal Guard | 120 | 8% [10/121] | 1.0 | 0.3 - 3.6 | 0.95 | 11% [13/121] | 0.8 | 0.3 - 2.3 | 0.67 | 17% [20/121] | 0.9 | 0.3 - 2.2 | 0.72 |  |
| Olyset Plus | 120 | 10% [13/124] | 1.0 | 0.3 - 3.5 | 0.89 | 18% [22/124] | 1.5 | 0.6 - 3.8 | 0.430 | 17% [ 21/124] | 0.9 | 0.3 - 2.3 | 0.87 |  |
|  |  |  |  |  |  |  |  |  |  |  |  |  |  |  |
