# Supplementary material for: Bio-efficacy of field aged novel class of long-lasting insecticidal nets, against pyrethroid-resistant malaria vectors in Tanzania: A series of experimental hut trials
Source: PLOS Glob Public Health. 2024 Oct 4;4(10):e0002586. doi: 10.1371/journal.pgph.0002586 (PMC11451999; doi:10.1371/journal.pgph.0002586)
Supplement: S2 Table — (DOCX) [file pgph.0002586.s005.docx]

| S2 Table: Percent mortality and blood feeding for *An. gambiae* s.l with their odds ratio and 95%CI. | | | | | | | | | | | | | | |
| --- | --- | --- | --- | --- | --- | --- | --- | --- | --- | --- | --- | --- | --- | --- |
|  |  | 24 hours mortality % (n/N) | | | | 72 hours mortality % (n/N) | | | | BF % (n/N) | | | | |
|  | Total collection | mortality % (n/N) | OR* | 95%CI | p value | mortality % (n/N) | OR* | 95%CI | p value | BF (n/N) | OR* | 95%CI | p value |  |
| **0 month** |  |  |  |  |  |  |  |  |  |  |  |  |  |  |
| Interceptor (reference) | 72 | 7 [ 5/73] | 1 |  |  | 11 [8/72] | 1 |  |  | 19 [14/73] | 1 |  |  |  |
| Interceptor G2 | 72 | 42 [28/66] | 12.4 | 3.1 - 49.2 | <0.001 | 55 [36/66] | 15.2 | 4.6 - 50.9 | <0.001 | 24 [16/66] | 1.5 | 0.5 - 5.0 | 0.504 |  |
| Royal Guard | 72 | 34 [ 27/79] | 10.1 | 2.7 - 37.8 | <0.001 | 37 [29/79] | 6.6 | 2.1 - 20.8 | <0.001 | 13 [10/79] | 0.6 | 0.2 - 1.9 | 0.37 |  |
| Olyset Plus | 72 | 44 [26/59] | 17.3 | 4.3 - 69.5 | <0.001 | 54 [32/59] | 13.1 | 3.9 - 43.7 | <0.001 | 12 [7/59] | 0.4 | 0.1 - 1.7 | 0.225 |  |
| **12 month** |  |  |  |  |  |  |  |  |  |  |  |  |  |  |
| Interceptor (reference) | 120 | 13 [16/128] | 1 |  |  | 22 [28/128] | 1 |  |  | 13 [ 17/128] | 1 |  |  |  |
| Interceptor G2 | 120 | 35 [ 54/155] | 4.9 | 2.1 - 11.8 | <0.001 | 42 [65/155] | 3.2 | 1.5 - 6.7 | 0.002 | 19 [ 29/155] | 1.2 | 0.5 - 3.0 | 0.685 |  |
| Royal Guard | 120 | 22 [ 36/161] | 2.5 | 1.0 - 6.0 | 0.04 | 34 [54/161] | 2.1 | 1.0 -4.3 | 0.058 | 11 [17/161] | 0.7 | 0.2 - 1.7 | 0.382 |  |
| Olyset Plus | 119 | 23 [35/155] | 2.6 | 1.0 - 6.4 | 0.045 | 30 [47/155] | 1.7 | 0.8 - 3.8 | 0.174 | 9 [ 14/155] | 0.4 | 0.1 - 1.1 | 0.086 |  |
| **24 month** |  |  |  |  |  |  |  |  |  |  |  |  |  |  |
| Interceptor (reference) | 120 | 9 [11/120] | 1 |  |  | 19 [23/120] | 1 |  |  | 23 [ 28/120] | 1 |  |  |  |
| Interceptor G2 | 120 | 18 [ 22/121] | 2.3 | 0.8 - 6.7 | 0.13 | 23 [28/121] | 1.3 | 0.5 - 3.2 | 0.578 | 28 [ 34/121] | 1.1 | 0.4 - 2.9 | 0.787 |  |
| Royal Guard | 120 | 13 [17/126] | 2.0 | 0.6 - 5.9 | 0.232 | 24 [30/126] | 1.6 | 0.7 - 4.0 | 0.298 | 35 [ 44/126] | 1.6 | 0.6 - 4.0 | 0.327 |  |
| Olyset Plus | 120 | 20 [19/97] | 2.5 | 0.8 - 7.7 | 0.125 | 24 [23/97] | 1.2 | 0.4 - 3.1 | 0.75 | 21 [ 20/97] | 0.8 | 0.3 - 2.2 | 0.672 |  |
| **36 month** |  |  |  |  |  |  |  |  |  |  |  |  |  |  |
| Interceptor (reference) | 120 | 11 [5/44] | 1 |  |  | 16 [7/44] | 1 |  |  | 25 [11/44] | 1 |  |  |  |
| Interceptor G2 | 120 | 9 [5/55] | 0.7 | 0.1 - 3.5 | 0.646 | 13 [7/55] | 0.7 | 0.2 - 2.9 | 0.658 | 13 [7/55] | 0.3 | 0.1 - 1.3 | 0.107 |  |
| Royal Guard | 120 | 8 [6/77] | 0.6 | 0.1 - 2.9 | 0.545 | 16 [12/77] | 0.9 | 0.3 - 3.5 | 0.982 | 21 [16/77] | 0.8 | 0.2 - 2.8 | 0.741 |  |
| Olyset Plus | 120 | 10 [7/67] | 0.8 | 0.2 - 3.7 | 0.791 | 22 [15/67] | 1.6 | 0.5 - 5.6 | 0.457 | 16 [11/67] | 0.5 | 0.1 - 1.8 | 0.295 |  |
|  |  |  |  |  |  |  |  |  |  |  |  |  |  |  |
|  |  |  |  |  |  |  |  |  |  |  |  |  |  |  |
