# Supplementary material for: Bio-efficacy of field aged novel class of long-lasting insecticidal nets, against pyrethroid-resistant malaria vectors in Tanzania: A series of experimental hut trials
Source: PLOS Glob Public Health. 2024 Oct 4;4(10):e0002586. doi: 10.1371/journal.pgph.0002586 (PMC11451999; doi:10.1371/journal.pgph.0002586)
Supplement: S3 Table — (DOCX) [file pgph.0002586.s006.docx]

| S3 Table: Percent mortality and blood feeding for *An. gambiae* s.l with their odds ratio and 95%CI. | | | | | | | | | | | | | | |
| --- | --- | --- | --- | --- | --- | --- | --- | --- | --- | --- | --- | --- | --- | --- |
|  |  | 24 hours mortality % (n/N) | | | | 72 hours mortality % (n/N) | | | | BF % (n/N) | | | | |
|  | Total collection | mortality % (n/N) | OR* | 95%CI | p value | mortality % (n/N) | OR* | 95%CI | p value | BF (n/N) | OR* | 95%CI | p value |  |
| **0 month** |  |  |  |  |  |  |  |  |  |  |  |  |  |  |
| Interceptor (reference) | 72 | 5 [2/38] | 1 |  |  | 18 [ 7/38] | 1 |  |  | 24 [ 9/38] | 1 |  |  |  |
| Interceptor G2 | 72 | 53 [ 19/36] | 56.0 | 6.2 - 504.1 | <0.001 | 56 [20/36] | 11.1 | 2.4 - 49.8 | 0.002 | 17[ 6/36] | 0.6 | 0.1 - 2.6 | 0.464 |  |
| Royal Guard | 72 | 10 [ 6/60] | 3.5 | 0.4 - 30.7 | 0.252 | 23 [ 14/60] | 1.6 | 0.4 - 6.6 | 0.499 | 8 [5/60] | 0.3 | 0.1 - 1.3 | 0.112 |  |
| Olyset Plus | 72 | 26 [14/53] | 13.4 | 1.7 - 108.5 | 0.015 | 37 [19/53] | 3.2 | 0.8 - 12.8 | 0.099 | 15 [ 8/53] | 0.5 | 0.1 - 2.1 | 0.36 |  |
| **12 month** |  |  |  |  |  |  |  |  |  |  |  |  |  |  |
| Interceptor (reference) | 120 | 13 [ 7/53] | 1 |  |  | 19 [10/53] | 1 |  |  | 21 [ 11/53] | 1 |  |  |  |
| Interceptor G2 | 120 | 37 [ 14/38] | 5.9 | 1.4 - 25.3 | 0.016 | 50 [ 19/38] | 5.1 | 1.5 - 17.8 | 0.01 | 11 [ 4/38] | 0.4 | 0.1 - 1.6 | 0.183 |  |
| Royal Guard | 120 | 16 [ 10/61] | 1.4 | 0.3 - 5.7 | 0.734 | 28 [ 17/61] | 1.9 | 0.6 - 6.2 | 0.27 | 11 [7/61] | 0.4 | 0.1 - 1.5 | 0.183 |  |
| Olyset Plus | 119 | 15 [ 15/97] | 1.2 | 0.3 - 4.6 | 0.811 | 21 [ 20/97] | 1.1 | 0.3 - 3.3 | 0.93 | 19 [18/97] | 0.8 | 0.3 - 2.6 | 0.738 |  |
| **24 month** |  |  |  |  |  |  |  |  |  |  |  |  |  |  |
| Interceptor (reference) | 120 | 14 [ 7/50] | 1 |  |  | 22 [ 11/50] | 1 |  |  | 20 [10/50] | 1 |  |  |  |
| Interceptor G2 | 120 | 17 [10 / 59] | 1.4 | 0.3 - 5.8 | 0.686 | 25 [ 15/59] | 1.3 | 0.4 - 4.3 | 652 | 25 [15/59] | 0.9 | 0.3 - 3.3 | 0.956 |  |
| Royal Guard | 120 | 14 [14/99] | 0.8 | 0.2 - 3.2 | 0.734 | 21 [ 21/99] | 0.9 | 0.3 - 2.7 | 0.806 | 35 [35/99] | 1.9 | 0.6 - 5.8 | 0.262 |  |
| Olyset Plus | 120 | 10 [11/111] | 0.7 | 0.2 - 2.8 | 0.6 | 23 [ 26/111] | 1.2 | 0.4 - 3.6 | 0.73 | 26 [29/111] | 1.3 | 0.4 - 3.9 | 0.655 |  |
| **36 month** |  |  |  |  |  |  |  |  |  |  |  |  |  |  |
| Interceptor (reference) | 120 | 2 [ 1/47] | 1 |  |  | 13 [6/47] | 1 |  |  | 13 [6/47] | 1 |  |  |  |
| Interceptor G2 | 120 | 13 [ 5/40] | 7.2 | 0.5 - 111.1 | 0.156 | 28 [ 11/40] | 2.9 | 0.6 - 13.4 | 0.181 | 18 [7/40] | 1.1 | 0.2 - 5.9 | 0.876 |  |
| Royal Guard | 120 | 5 [2/44] | 1.7 | 0.1 - 32.7 | 0.728 | 5 [2/44] | 0.2 | 0.02 -1.7 | 0.136 | 9 [4/44] | 0.6 | 0.1 - 3.5 | 0.604 |  |
| Olyset Plus | 120 | 4 [2/56] | 1.6 | 0.1 - 26.6 | 0.754 | 12 [7/56] | 0.9 | 0.2 - 4.3 | 0.921 | 19 [11/56] | 1.5 | 0.3 - 6.4 | 0.584 |  |
