# Supplementary material for: Bio-efficacy of field aged novel class of long-lasting insecticidal nets, against pyrethroid-resistant malaria vectors in Tanzania: A series of experimental hut trials
Source: PLOS Glob Public Health. 2024 Oct 4;4(10):e0002586. doi: 10.1371/journal.pgph.0002586 (PMC11451999; doi:10.1371/journal.pgph.0002586)
Supplement: S4 Table — (DOCX) [file pgph.0002586.s007.docx]

S4 Table: Total collected mosquitoes per treatment per time point inside net and exit traps with percent deterrence

|  | ***An.gambiae* s.l.** | | | |  | ***An.funestus* s.l.** | | | |
| --- | --- | --- | --- | --- | --- | --- | --- | --- | --- |
| **Treatment** | **Total Caught** | **% Deterrence** | **% Exit (95%CI)** | **% Net (95%CI)** |  | **Total Caught** | **% Deterrence** | **% Exit (95%CI)** | **% Net (95%CI)** |
| **0 month** |  |  |  |  |  |  |  |  |  |
| Untreated net | 63 | ref | 68 (58 - 79) | 11 (4 - 18) |  | 41 | ref | 68 ( 54 - 83) | 17 (4 - 30) |
| Interceptor | 75 | -19% | 81 (71 - 91) | 1 ( 0 - 4) |  | 38 | 7% | 76 ( 63 - 90) | 8 ( 0 - 15) |
| InterceptorG2 | 66 | -5% | 67 (56 - 78) | 9 ( 3 - 15) |  | 36 | 12% | 61 ( 39 - 84) | 11 ( 1 - 21) |
| Royal Guard | 79 | -25% | 78 (68 - 89) | 3 ( 0 - 6) |  | 60 | -46% | 77 ( 61 - 93) | 3 ( 0 - 8) |
| Olyset Plus | 59 | 6% | 71 (52 - 90) | 5 ( 0 - 11) |  | 53 | -29% | 74 (63 - 85) | 6 ( 0 - 12) |
| 12 months |  |  |  |  |  |  |  |  |  |
| Untreated net | 176 | ref | 65 (57 - 73) | 22 (16 - 28) |  | 54 | ref | 54 ( 38 - 70) | 20 (7 - 33) |
| Interceptor | 128 | 27% | 82 ( 74 - 90) | 5 ( 1 - 8) |  | 53 | 2% | 89 ( 80 - 98) | 2 ( 0 - 6) |
| Interceptor G2 | 155 | 12% | 72 (63 - 82) | 7 ( 2 - 12) |  | 38 | 30% | 74 ( 58 - 89) | 3 ( 0 - 8) |
| Royal Guard | 161 | 9% | 79 (71 - 86) | 5 ( 0 - 9) |  | 61 | -13% | 85 ( 77 - 94) | 3 ( 0 - 8) |
| Olyset Plus | 153 | 13% | 79 ( 72 - 86) | 5 ( 1 - 9) |  | 97 | -80% | 82 ( 73 - 92) | 1 ( 0 - 3) |
| 24 months |  |  |  |  |  |  |  |  |  |
| Untreated net | 78 | 1 | 68 (58 - 78) | 13 (5 - 20) |  | 81 | 1 | 59 (45 - 73) | 23 (13 - 34) |
| Interceptor | 120 | -54% | 74 ( 65 - 84) | 6 ( 1 - 10) |  | 50 | 38% | 80 ( 69 - 91) | 8 ( 0 - 16) |
| Interceptor G2 | 121 | -55% | 65 (55 - 75) | 14 ( 7 - 22) |  | 59 | 27% | 73 ( 55 - 91) | 8 ( 1 - 16) |
| Royal Guard | 126 | -62% | 60 (51 - 70) | 4 ( 0 - 9) |  | 99 | -22% | 79 ( 68 - 89) | 4 ( 0 -9) |
| Olyset Plus | 97 | -24% | 75 ( 66 - 85) | 1 ( 0 - 3) |  | 111 | -37% | 84 ( 76 - 91) | 2 ( 0 - 4) |
| 36 months |  |  |  |  |  |  |  |  |  |
| Untreated net | 81 | 1 | 57 (45 - 69) | 11 (5 - 17) |  | 52 | 1 | 69 (55 - 84) | 12 (3 - 20) |
| Interceptor | 44 | 46% | 73 (57 - 88) | 2 (0 - 7) |  | 47 | 10% | 57 ( 38 - 77) | 13 ( 0 - 24) |
| InterceptorG2 | 55 | 32% | 62 (50 - 74) | 5 (0 - 11) |  | 40 | 23% | 65 ( 44 - 86) | 13 ( 0 - 30) |
| Royal Guard | 77 | 5% | 78 (68 - 87) | 1 ( 0 - 4) |  | 44 | 15% | 84 (75 - 93) | 0 |
| Olyset Plus | 67 | 17% | 85 (77 - 93) | 1 (0 - 4) |  | 57 | -10% | 79 ( 65 - 92) | 4 ( 0 - 8) |
