# Supplementary material for: Bio-efficacy of field aged novel class of long-lasting insecticidal nets, against pyrethroid-resistant malaria vectors in Tanzania: A series of experimental hut trials
Source: PLOS Glob Public Health. 2024 Oct 4;4(10):e0002586. doi: 10.1371/journal.pgph.0002586 (PMC11451999; doi:10.1371/journal.pgph.0002586)
Supplement: S5 Table — (DOCX) [file pgph.0002586.s008.docx]

S5 Table: Effect of hole on blood feeding in Anopheles mosquitoes collected.

| Net age |  | Interceptor G2 | | | | | Olyset Plus | | | | | Royal Guard | | | | |
| --- | --- | --- | --- | --- | --- | --- | --- | --- | --- | --- | --- | --- | --- | --- | --- | --- |
| 12 | Hole area (per cm2) | mean hole area | Blood feeding | | | | mean hole area | Blood feeding | | | | mean hole area | Blood feeding | | | |
|  |  |  | % (n/N) | OR* | 95%CI | p value |  | % (n/N) | OR* | 95%CI | p value |  | % (n/N) | OR* | 95%CI | p value |
|  | < 79 | 26.5 | 13.8 (17/123) | 1 |  |  | 27.9 | 7.1(3/42) |  |  |  | 16.2 | 8 (9/107) |  |  |  |
|  | 80 to 789 | 242.5 | 20.8 (10/48) | 1 | 0.3 - 3.1 | 0.962 | 390.8 | 10.9(8/73) | 0.6 | 0.2 - 1.6 | 0.319 | 260.3 | 12.7 (8/63) | 0.9 | 0.4 - 2.5 | 0.968 |
|  | >790 | 1744.8 | 27.3(6/22) | 1.5 | 0.4 - 1.6 | 0.592 | 3243.8 | 15.5(21/135) | 1.2 | 0.4 - 3.3 | 0.714 | 1641.1 | 13.5 (7/52) | 1.2 | 0.4 - 3.6 | 0.802 |
|  | | | | | | | | | | | | | | | | |
| 24 | < 79 | 28.7 | 27.0 (10/37) |  |  |  | 29.8 | 11.1 (2/18) |  |  |  | 19.8 | 32.2 (28/87) |  |  |  |
|  | 80 to 789 | 255.2 | 22.7 (10/44) | 0.6 | 0.2 - 1.7 | 0.306 | 475.7 | 8.3 (2/24) | 0.4 | 0.1 - 1.6 | 0.197 | 530.5 | 0 (0/3) | 0.5 | 0.2 - 1.6 | 0.272 |
|  | >790 | 2064.5 | 29.3 (29/99) | 0.6 | 0.2 - 1.7 | 0.369 | 2890.1 | 27.1 (45/166) | 1.1 | 0.4 - 3.5 | 0.806 | 2587.8 | 37.8 (51/135) | 1.1 | 0.6 - 2.1 | 0.813 |
|  | | | | | | | | | | | | | | | | |
| 36 | < 79 | 35.4 | 11.1 (2/18) |  |  |  | 17.2 | 27.3 (3/11) |  |  |  | 35.9 | 14.3 (3/21) |  |  |  |
|  | 80 to 789 | 402.6 | 23.3 (7/30) | 4.1 | 0.2 - 75.4 | 0.34 | 305.7 | 3.8 (1/26) | 0.6 | 0.1 - 5.3 | 0.614 | 397.8 | 14.3 (4/28) | 1.9 | 0.3 - 12.4 | 0.493 |
|  | >790 | 1951.6 | 10.6 (5/47) | 0.7 | 0 - 12.9 | 0.822 | 3592.9 | 20.7 (18/87) | 0.6 | 0.1 - 4.9 | 0.672 | 2430.6 | 18.1 (13/72) | 1.5 | 0.3 - 8.4 | 0.626 |
